# CCL5 Neutralization Restricts Cancer Growth and Potentiates the Targeting of PDGFRβ in Colorectal Carcinoma

**DOI:** 10.1371/journal.pone.0028842

**Published:** 2011-12-20

**Authors:** Béatrice Cambien, Peggy Richard-Fiardo, Babou F. Karimdjee, Violette Martini, Bernard Ferrua, Bruno Pitard, Heidy Schmid-Antomarchi, Annie Schmid-Alliana

**Affiliations:** 1 Université de Nice Sophia Antipolis, UFR Médecine, Nice, France; 2 INSERM UMR 576, Nice, France; 3 Clinique St Jean, Cagnes sur mer, France; 4 INSERM U895, Nice, France; 5 INSERM U915, Nantes, France; 6 In-Cell-Art, Nantes, France; University of Missouri-Columbia, United States of America

## Abstract

Increased CCL5 levels are markers of an unfavourable outcome in patients with melanoma, breast, cervical, prostate, gastric or pancreatic cancer. Here, we have assessed the role played by CCL5/CCR5 interactions in the development of colon cancer. To do so, we have examined a number of human colorectal carcinoma clinical specimens and found CCL5 and its receptors over-expressed within primary as well as liver and pulmonary metastases of patients compared to healthy tissues. In vitro, CCL5 increased the growth and migratory responses of colon cancer cells from both human and mouse origins. In addition, systemic treatment of mice with CCL5-directed antibodies reduced the extent of development of subcutaneous colon tumors, of liver metastases and of peritoneal carcinosis. Consistently, we found increased numbers of CD45-immunoreactive cells within the stroma of the remaining lesions as well as at the interface with the healthy tissue. In contrast, selective targeting of CCR5 through administration of TAK-779, a CCR5 antagonist, only partially compromised colon cancer progression. Furthermore, CCL5 neutralization rendered the tumors more sensitive to a PDGFRβ-directed strategy in mice, this combination regimen offering the greatest protection against liver metastases and suppressing macroscopic peritoneal carcinosis. Collectively, our data demonstrate the involvement of CCL5 in the pathogenesis of colorectal carcinoma and point to its potential value as a therapeutic target.

## Introduction

Tumor-stroma interactions are recognized as critical components of tumor invasion and metastatic potential of colon carcinoma [1]. Stromal, inflammatory and cancer cells communicate among themselves directly through cell contact but also indirectly through paracrine signals [2], [3]. Such signals favor tumor development in multiple ways: they act as growth factors, stimulate angiogenesis, modulate the extracellular matrix, induce the recruitment of additional stromal cells and take part in immune evasion mechanisms of cancer. As a consequence, identification of tumor-promoting factors for cancer therapeutics has become of major interest to devise anti-tumor strategies to be applied either as single-agent treatment or as combination therapy in case where tumors fail to respond to monotherapy. Various factors have been identified so far as promoters of colon cancer progression, most common of which are the VEGF (vascular endothelial growth factor) family, the FGF (fibroblast growth factor) family and the PDGF (platelet-derived growth factor) family, their production within the neoplasm correlating with tumor grade and shorter patient survival [4]–[8]. More recently, there has been increasing evidence from various studies including ours that the chemokines produced within the tumor microenvironment may also play a crucial role in the pathogenesis of CRC (colorectal carcinoma) [9]–[12].

Among the chemokines thought to strongly promote carcinogenesis and stromagenesis is CCL5/RANTES (CC chemokine ligand 5/Regulated upon activation, normal T-cell-expressed and secreted) which was initially described for its key role in inflammatory diseases. Indeed, clinical evidence has revealed that elevated levels of tissue or plasma CCL5 are markers of an unfavourable outcome in patients with either melanoma, breast, cervical, prostate, gastric or pancreatic cancer [13]–[20]. In breast cancer, *in vivo* CCL5 neutralization or CCR5 antagonism were shown to abrogate the MSC-induced metastasis of cancer cells thus implicating CCL5/CCR5 as a key axis in this malignancy [21]. Selective targeting of the CCR5/CCL5 signaling also led to reduced tumor growth in experimental pancreatic adenocarcinoma through disruption of CCR5-dependent recruitment of regulatory T cells into tumors [22]. Anibamine, a new CCR5 antagonist also suppressed the invasive and metastatic properties of prostate cancer cells in mice [23]. Finally, CCL5 blockade significantly compromised gastric cancer progression [20]. Interestingly, CCL5 has recently been reported to be expressed in colorectal carcinoma, predominantly at the invasive front of primary tumors [24]. Based on the aforementioned clinical observations in several cancers, it is tempting to speculate that CCL5 and its receptors may have a substantial role in CRC progression and may thus represent an interesting target for the treatment of this malignancy. To date, however, none of these aspects have been addressed *in vivo*.

The study described herein aimed precisely at getting new insights into the role played by CCL5/CCR5 interactions in the development of colorectal carcinoma. To do so, we have examined a number of human colon cancer clinical specimens and found CCL5 and its receptors over-expressed within primary as well as liver and pulmonary metastases of CRC patients compared to healthy tissues. To assess the relevance of CCL5/CCR5 neutralization in colon carcinoma, we have used syngeneic experimental models of orthotopic (liver) and ectopic (subcutis) colon cancer in immunocompetent mice. We have examined the effect of CCL5- or CCR5-blockers administered as single agents or in combination with a murine PDGFRβ-directed treatment, this strategy being currently under clinical evaluation for the treatment of CRC cancers. This report provides the first preclinical evidence for a role of CCL5 in colon carcinoma and demonstrates that CCL5 blockade has the potential to reduce colon cancer progression and to improve the therapeutic response in multidrug regimen.

## Materials and Methods

### Reagent

The following reagent (TAK-779) was obtained through the NIH AIDS Research and Reference Reagent Program, Division of AIDS, National Institute of Allergy and Infectious Diseases, NIH.

### Tumor Cell lines and Experimental Animals

Human HT29 and mouse CT26 colon carcinoma cells were purchased from ATCC and maintained in DMEM medium supplemented with 10% heat-inactivated FBS as previously described [11]. Female SCID and BALB/c mice, 6 to 8 weeks old, were purchased from Harlan (Gannat, France).

### Ethics

Ten sets of primary colon cancer tumors and metastatic tissues from the same patients as well as paired “healthy” colonic biopsies were collected from patients with invasive adenocarcinomas, who underwent surgical biopsies or initial surgery at the Institut Paoli-Calmettes (IPC, Marseille, France) between 1987 and 2007. Each patient gave written informed consent and the study was approved by the IPC “Comité d'Orientation Stratégique”. All of the procedures involving laboratory animals and their care were approved by institutional review board (Permit number # A06-088-14).

### TaqMan real-time PCR experiments

Total RNA from human colorectal cancer and healthy colon, from mouse healthy and tumor tissues as well as colon carcinoma cell lines was extracted using RNeasy kit (Qiagen, Courtaboeuf, France) and transcribed into cDNA using the Superscript III enzyme (Invitrogen, Cergy Pontoise, France) as previously described [11]. Real-time PCR was performed in an ABI PRISM 7900HT and carried out using TaqMan® gene expression assays for human samples (hCCR1: Hs 00174298m1, hCCR3: Hs 00356601m1, hCCR5 Hs 00152917m1, hCCL5: Hs 00174575m1) (Applied Biosystem, Courtaboeuf, France), or SYBR® gene expression assays according to the manufacturer's instructions (Applied Biosystem). The primer sequences for mouse CCL5 were: forward, 5′- TGCCCACGTCAAGGAGTATTTC-3′; and reverse, 5′- AACCCACTTCTTCTCTGGGTTG-3′, for mouse CCR1: forward, 5′- AGGCCCAGTGGGAGTTCAC-3′; and reverse, 5′- TCTTCCACTGCTTCAGGCTCTT-3′, for mouse CCR3: forward, 5′- AAGCTTTGAGACCACACCCTATG-3′; and reverse, 5′- GACCCCAGCTCTTTGATTCTGA-3′; for mouse CCR5, forward 5′- TTATCTCTCAGTGTTCTTCCGAAAAC-3′ and reverse, 5′- TTCTCCTGTGGATCGGGTATAGA-3′. Relative levels in mRNA expression were determined using ΔC_T_ values obtained by subtracting C_T_ control (human or mouse actin) from C_T_ target gene measured in the same RNA preparation. Comparative level of mRNA expression between healthy (*X*) and metastatic tissues (*Y*) was calculated using the formula Δ*C*_T_ *Y* – *ΔC*_T_ *X* and expressed as fold over healthy (2ΔΔ*C*_T_). Murine healthy colon tissues were used to analyse mouse tumors and human healthy colon specimens were used to analyse human tumors and HT-29 samples.

### *In vitro* proliferation assay

Briefly, colon cancer cells pretreated or not with TAK-779 or anti-CCL5 antibodies (at the indicated concentrations) were seeded at a density of 10^4^ cells/cm^2^ and incubated either in serum-enriched medium or in base medium (containing 0.1% Bovine Serum Albumin) supplemented or not with various concentrations of recombinant CCL5 (Peprotech, Neuilly sur Seine, France) for 5 days before being trypsin-detached, collected and enumerated as previously described [11].

### *In vitro* chemotaxis assay

Chemotactic responses of colon cancer cells were evaluated by using 24-well chemotaxis chambers and polyethylene terephtalate inserts with 8 µm pores (Becton Dickinson, San Jose, CA) coated with 6.5 µg/mL fibronectin (Sigma, Lyon, France) or with 50 µg/mL collagen (Becton Dickinson) for the CT26 cells or the HT29 cells, respectively [11]. Colon cancer cells, pretreated or not with TAK-779 or anti-CCL5 antibodies (at the indicated concentrations), were placed in the upper well (5×10^4^ cells) and various concentrations of recombinant CCL5 (Peprotech) were added to the lower wells. After incubation of the plates for 18 hours (CT26 cells) or for 40 hours (HT29 cells) at 37°C in 5% CO_2_ atmosphere, non-migrated cells were removed from the upper well and the migrated cells collected on the lower side of the insert were stained using crystal violet dye and enumerated. Migration index was calculated as the ratio of the number of migrated cells in chemoattractant-containing wells divided by the number of cells that migrated to base medium alone.

### PDGFRβ gene expression vector

DNA encoding mPDGFRβ was cloned and inserted into the pTOPO-cDNA3 eukaryotic expression vector (Invitrogen). Identities of sense (pcDNA3-PDGFRβ) and antisense (control vector) products were confirmed by sequencing.

### Plasmid preparation and formulation

The DNA vaccine plasmid (pcDNA3-PDGFRβ) and the corresponding control vector were used as antigens. All plasmids were purified using EndoFree plasmid purification columns (Qiagen) and were confirmed to be free of endotoxin contamination (endotoxin <0.1 EU/µg plasmid DNA) by the Limulus amoebocyte lysate assay (Lonza, Le Perray en Yvelines, France). The tetrafunctionalized amphiphilic block copolymer 704 (MW 5,500) was supplied by In-Cell-Art (Nantes, France). Stock solutions were prepared at 20% (w/v) in water and stored at 4°C. Formulations of DNA with 704 (final concentration 0.3%) were prepared immediately prior to intramuscular injection by equivolumetric mixing of copolymers in water with plasmid DNA solution as previously described [25], [26]. DNA doses administered are indicated throughout the study.

### Transfection with PDGFRβ gene expression vector

The correct expression of mPDGFRβ was verified by transient transfection of CHO cells with the control vector and the DNA vaccine plasmid (pcDNA3-PDGFRβ) using AMAXA Biosystems (Lonza). Twenty-four hours post-transfection, cell lysates from CHO cells were prepared as previously described [27] before being subjected to SDS-PAGE. Western blotting was performed either with goat anti-mouse PDGFRβ antibodies (Santa Cruz Biotechnology, Le Perray-en-Yvelines, France) or with diluted serum obtained from the PDGFRβ-vaccinated- mice. Detection was done either by rabbit anti-goat Abs- or by goat-anti mouse Abs- conjugated to horseradish peroxidase (Dako, Trappes, France). Bands were visualized by chemiluminescence-enhanced reaction (Amersham, Les Ulis, France).

### Mouse models

#### Subcutaneous, liver and pulmonary metastasis models

For the induction of subcutaneous and hepatic tumors, CT26 cells (5×10^4^) or HT29 cells (3×10^6^) were injected in the flank or under the liver capsule of BALB/c or SCID mice, respectively. For the induction of pulmonary metastases, CT26 cells (3×10^4^) or HT29 cells (2×10^6^) were delivered by intravenous tail injection into BALB/c or SCID mice, respectively. At sacrifice, complete post-mortem examinations were performed. Subcutaneous and hepatic tumors were excised, weighed and measured with callipers in the two perpendicular axes (*a* and *b*). Tumour volume was calculated according to the formula *ab* ^2^π/6.

#### Intramuscular DNA vaccination

Following a prophylactic setting, anesthetized mice were vaccinated 3 times at 3-week interval (days 0, 21 and 42) by intramuscular injections of DNA-polymer formulations into both *tibialis anterior* muscles as previously described [25], [26]. Three weeks after the last boost (day 66), mice were challenged by injection of 5×10^4^ CT26 murine colon carcinoma cells under the liver capsule.

#### Mouse treatments

Anti-CCL5 or control isotype IgG antibodies (32 µg per mouse, Peprotech) were injected into the peritoneum of BALB/c mice 72 h after tumor implantation and twice weekly for the duration of the experiments (from day 69 to 87). TAK-779, the small-molecule CC chemokine receptor 5 (CCR5) inhibitor [28], [29] was injected into mice (150 µg/mouse) 72 h after tumor cells inoculation and daily thereafter until sacrifice (from day 69 to 87).

At sacrifice (day 87), hepatic tumors were excised and weighed. Incidence of peritoneal carcinosis into mice was expressed as percent of carcinosis-bearing animals in each group.

### Determination of serum levels of mPDGFRβ-specific Abs by ELISA

Serum was collected by retro-orbital bleeds at different time points after vaccination from immunized mice. ELISA plates were coated with 2 µg/ml anti-mouse PDGFRβ antibodies (Santa Cruz) in 100 µl PBS overnight at 4°C. The next morning, the plates were washed twice with PBS/Tween 20 (PBST), saturated for 30 minutes with phosphate buffer containing 1% skim milk and 0.12% Triton X-100 (assay buffer), washed again twice before being incubated for 18 h at room temperature with recombinant mouse PDGFRβ. After repeated washes, serial dilutions of serum samples from immunized mice were added to the well in duplicates. The plates were further incubated for 2 h, washed four times with PBST, and bound-enzyme activity was revealed with a chromogenic OPD substrate solution and measured at 490 nm.

### Histology/Immunohistochemistry

Formalin-fixed, paraffin-embedded sections of colon cancer metastatic tissues were stained with haematoxylin/eosin for morphologic evaluation. Immunostaining of CD45 was performed with anti-mouse CD45 mAb (BD Pharmingen, Le Pont de Claix, France) by the avidin-biotin complex immunoperoxidase method following microwave antigen retrieval. The primary antibody was replaced with isotype-matched antibodies in adjacent tissue sections as negative control. Expression of CD45 in mouse spleen was used as positive control.

### Statistical analysis

Results are expressed as mean ± s.e.m. and analyzed using the unpaired Student's *t* test or the Kruskal-Wallis test for multiple comparisons.

## Results

### Expression of CCL5 and its cognate receptors in the resected colorectal carcinoma specimens

**Figure 1:**
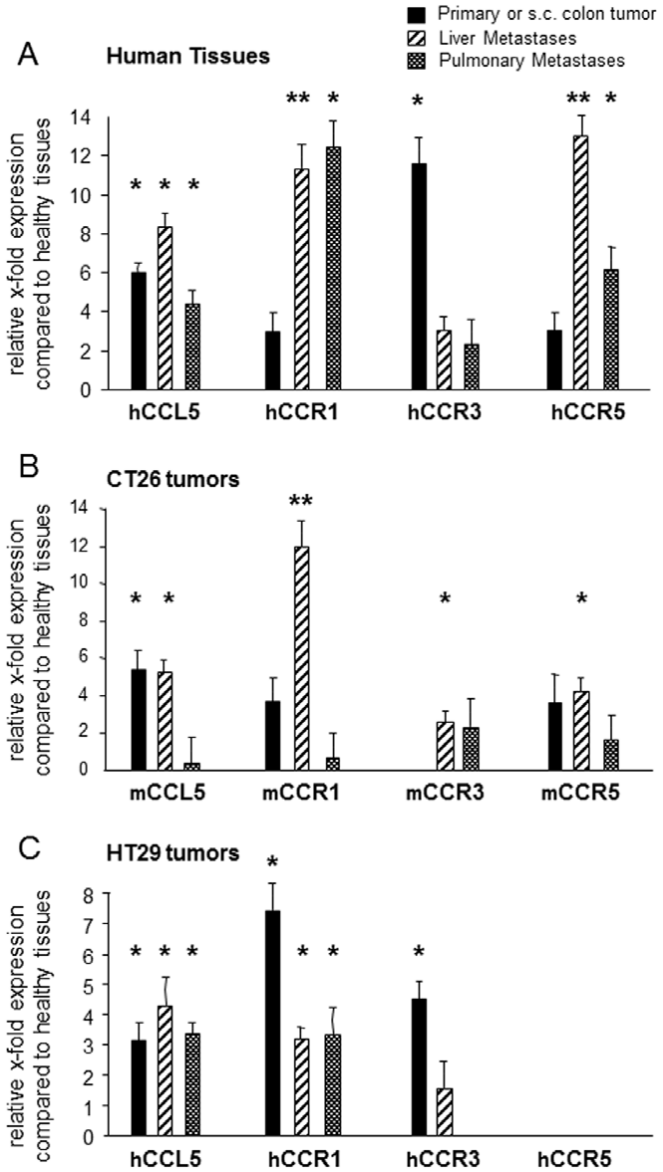
Expression of CCL5 and its corresponding CCR1, CCR3, CCR5 receptors in human biopsies and mouse tissues of colorectal carcinoma. Analysis of levels of expression of human (h) or mouse (m) targets was performed by quantitative RT-PCR in surgical resection pieces of human colorectal carcinoma (n = 10) (A), in experimental subcutaneous tumors (n = 6), liver (n = 6) and lung metastases (n = 6) derived from mouse CT26 cells (B) or derived from human HT29 cells (C) compared with corresponding healthy tissues (normal human, n = 10, and normal mouse colon, n = 6, respectively). The relative levels of expression were calculated using standard curves and expressed as 1/ΔCt. ΔCt values were calculated by substracting Ct of normalizing gene from Ct of target gene, measured in the same RNA preparation. Comparative level of mRNA expression between healthy (*X*) and metastatic tissues (*Y*) was calculated using the formula Δ*C*_T_ *Y* – Δ*C*_T_ *X* and expressed as fold over healthy (2ΔΔ*C*_T_). Black bars: primary or subcutaneous colon tumors; hatched bars: liver metastases; dotted bars: lung metastases. * p<0.05, ** p<0.01.

We analysed by quantitative RT-PCR (real time-quantitative reverse transcription polymerase chain reaction) the expression levels of human CCL5 and its receptors CCR1, CCR3 and CCR5 on surgical resection pieces of human primary colon tumors, of paired hepatic and pulmonary colorectal cancer metastases and of corresponding healthy tissues collected from the same patients. We observed increased levels of CCL5 expression in colorectal tumors (6 fold, P<0.05), liver metastases (8.5 fold, P<0.05) and lung metastases (4 fold, P<0.05) compared to healthy specimens (Figure 1A, Table S1). Because CCL5 acts through specific receptors, we also looked for such receptors that were expressed within tumor and metastatic specimens. Distinct patterns of CCRs expression were measured according to the target organ. Whereas CCR1 and CCR5 were both found to be significantly overexpressed in malignant liver and lung tissues compared to corresponding control biopsies (P<0.01 in liver, P<0.05 in lungs), CCR3 was only found significantly overexpressed within primary colorectal tumors compared to healthy organs (P<0.05) (Figure 1A, Table S1).

In order to further assess the role of the CCL5/receptors axis in colon cancer, we have looked for a relevant mouse colon carcinoma model. To do so, we have analysed by quantitative RT-PCR the expression level of CCL5/CCRs in the human HT29 and in the mouse CT26 colon cancer cells grown in culture. We observed CCL5 and CCR5 expression by both cell lines in vitro (Table S1). None of the two other CCL5 receptors (CCR1 and CCR3) were detected in the HT29 cells whereas CCR1 expression was found in the CT26 cells. To analyse whether the expression patterns of CCL5/receptors within malignant tissues were in accordance with those observed in human biopsies, we next developed experimental models of orthotopic (liver and lung metastases) and ectopic (subcutis) colon cancer in immunocompetent and immunodeficient mice. Colon cancer cells of mouse (CT26) or human (HT29) origins were inoculated into mice either subcutaneously, under the liver capsule or through tail vein injection to generate pulmonary metastases. At sacrifice, levels of chemokines/receptors within the distinct target organs were evaluated by real-time quantitative PCR using murine primers for the CT-26-derived tmors and human primers for the HT-29 xenografts (Figure 1B and C). Elevated levels of CCL5 expression were detected in all tumor models (subcutaneous, hepatic and lung) developed with mouse CT26 cells and with human HT29 cells except in pulmonary lesions derived from CT26 cells. In contrast, the patterns of expression of CCL5 receptors were complex and changed when cells were grown in vivo compared to culture conditions. None of the two tumor models (CT26 and HT29) exactly reflected the pattern of chemokine receptors expression observed in the human biopsies. Therefore, based on the fact that all three CT26 tumor types (subcutaneous, liver and lung) expressed both CCL5 and CCR5 in vivo, whereas HT29 xenografts exhibited levels of CCR5 below the threshold of detection of the PCR technique, we have found more appropriate to work with the CT26 models to assess the role played by CCL5/CCR5 interaction in colorectal carcinoma. In addition, the CT26 syngeneic model that is developed into immunocompetent mice also appeared the most appropriate to take into account CCL5/CCR5-dependent immune mechanisms especially considering that our ultimate goal was to combine CCL5 blockade with a vaccine strategy.

### CCL5 enhances CRC cell proliferation and migration *in vitro*

**Figure 2:**
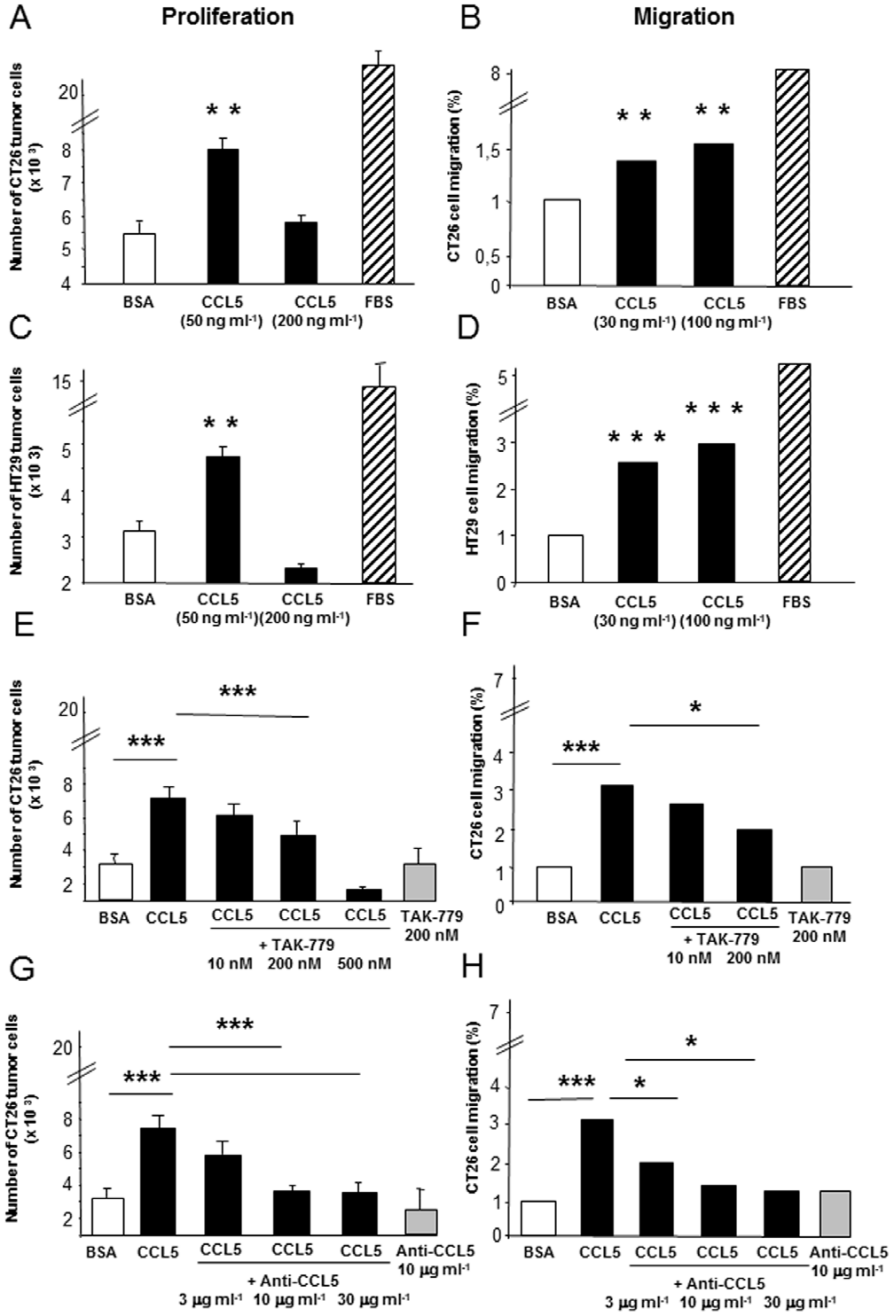
CCL5-induced tumor-promoting properties in CRC cells. (A, C, E, G) Proliferation of the CT26 and HT29 cells was assessed in response to a 5-day treatment with base medium alone (BSA, open bars), with serum-enriched medium (FBS, hatched bars) or with the indicated concentrations of recombinant CCL5 (filled bars), in the presence or in the absence of TAK-779 or anti-CCL5 antibodies (at the indicated concentrations). (B, D, F, H) CRC cells were assayed for chemotaxis in response to base medium alone (BSA, open bars), to serum-enriched medium (FBS, hatched bars) or to the indicated concentrations of recombinant CCL5 (filled bars), in the presence or in the absence of TAK-779 or anti-CCL5 antibodies. Results represent the mean±s.e.m. of six determinations. * p<0.05, ** p<0.01, *** p<0.001.

The expression of CCL5 receptors by human and murine colon carcinoma cells has led us to determine the ability of their common ligand CCL5 to stimulate their proliferation and migration *in vitro*. To this end, we examined and quantified cell growth after plating CT26 and HT29 cells for 5 days at low density in base medium alone or supplemented with various concentrations of CCL5. Within 5 days of serum starvation, we observed that CRC cells of both origins proliferate in response to CCL5 treatment in a dose-dependent manner (Figure 2A and C). The maximum growth was observed in response to 50 ng/ml CCL5. We next assessed the ability of the CRC cells to migrate in response to CCL5. CT26 and HT29 cells were harvested, placed into modified Boyden chambers and allowed to migrate towards various concentrations of CCL5. Figure 2B and D shows that CRC cells migrated to the chemokine compared with base medium alone. Given the key role played by CCR5 in mediating CCL5 actions in various cancer cells, we next attempted to disrupt the CCL5/CCR5 action using TAK-779, a non-peptide CCR5 antagonist previously described to impair tumor development in pancreatic cancer [22]. Increasing doses of TAK-779 (ranging from 10 to 500 nM) were tested on the CT26 cells according to IC50 values described in the low nM range [28], [29]. We observed a dose-dependent effect of TAK-779 on both the growth and the migratory responses of the CRC cells, as depicted on Figure 2E and F. The strongest inhibition was obtained with 200 nM TAK-779, higher doses leading to cytotoxic effects on the cells. However, only 28% of the CRC responses were abrogated by CCR5 blockade, thus suggesting the involvement of CCR5-independent mechanisms in both cellular processes. Various concentrations of anti-CCL5 antibodies, ranging from 3 to 30 µg/ml, were then applied to CT26 cells. As shown on Figure 2G and H, CCL5 neutralization obtained with the 10 µg/ml dose of antibodies totally impaired CRC cells migratory and growth responses to the chemokine. Collectively, these data suggest that CCL5/receptors activation appears as a common feature of the CRC cells from distinct origins and that it could mediate the malignancy-related properties of colon cancer cells.

### CCL5-CCRs interactions are involved in colon cancer development

**Figure 3:**
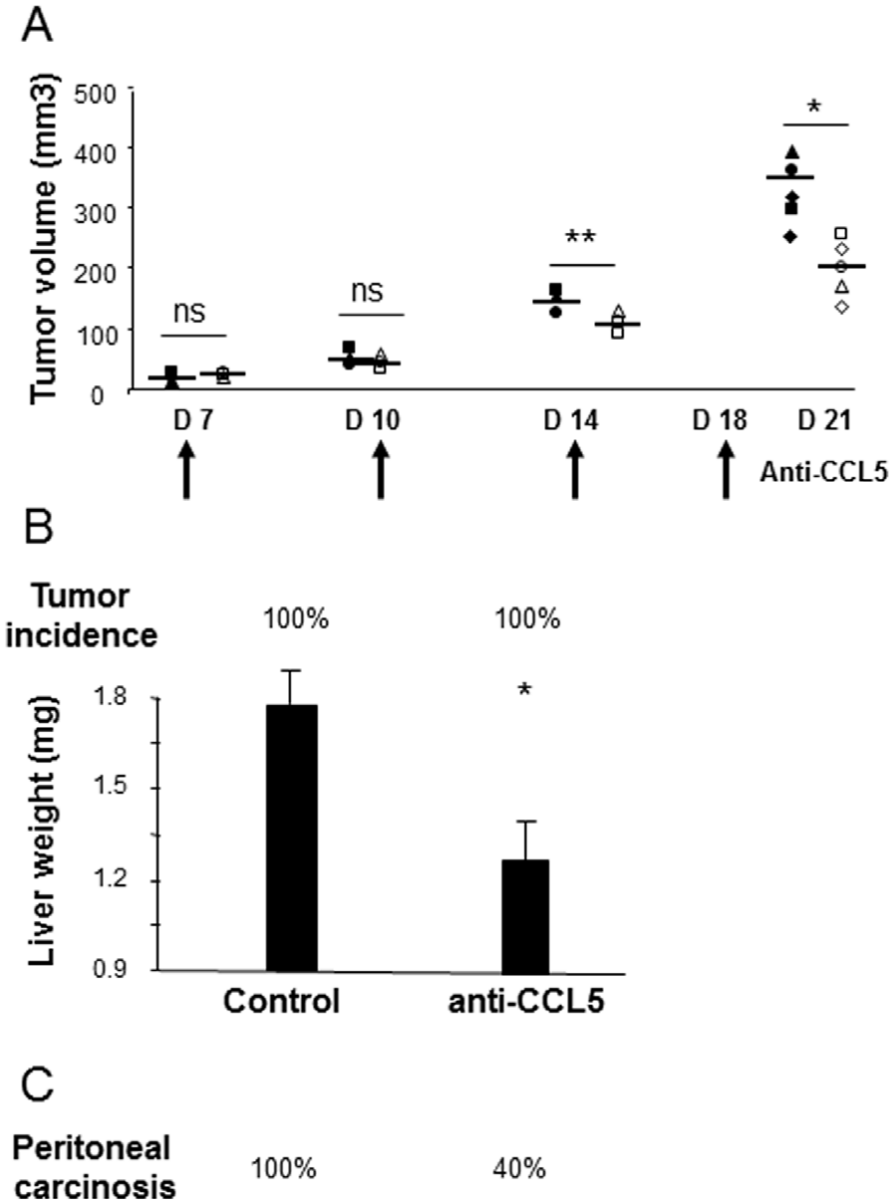
Protective effect of CCL5 neutralization on the development of colorectal tumors, metastases and peritoneal carcinosis. (A) Mice subcutaneously-challenged with CT26 cells received four intratumor injections of anti-CCL5 (open dots) or isotype-matched antibodies (filled dots) at the indicated times. Upon sacrifice, the extent of tumor development was assessed by measuring the tumor burdens. (B) Incidence and extent of tumor development within livers of CT26-challenged mice treated with anti-CCL5- or isotype-matched antibodies. (C) Incidence of peritoneal carcinosis expressed as percent of mice bearing carcinosis. (n = 5/group). * p<0.05, ** p<0.01.

To probe whether the malignancy-related properties of CCL5 exerted onto colon cancer cells *in vitro* also have a relevance *in vivo*, we evaluated the impact of CCL5 neutralization on the development of CT26 colon tumors implanted subcutaneously into immunocompetent Balb/c mice. At day 0, mice were challenged with a subcutaneous injection of CT26 cells under the skin. Animals were then treated on days +7, +10, +14 and +18 with intratumor administrations of anti-CCL5 or control isotype IgG antibodies. At sacrifice, the extent of tumor development was assessed by measuring the tumor burdens. CT26-challenged mice developed within 7 days a palpable tumor averaging 20 mm^3^ that grew to a size of ∼336 mm^3^ by day 21 (Figure 3A). In contrast, the anti-CCL5-treated mice developed tumors that grew with a reduced rate compared to control tumors, this reduction being clearly detectable after the third anti-CCL5 treatment (40% reduction, P<0.01). At sacrifice, their volume reached an average size of 202 mm^3^, corresponding to a 40% reduction compared to control burdens (P<0.05).

Given that liver is a major target organ in the CRC malignancy and that the highest expression levels of CCL5, CCR1 and CCR5 were found within liver metastases of human colon cancer patients, we next sought to assess the protective potential of the anti-CCL5 treatment on the development of experimental hepatic metastases into immunocompetent mice. Animals were thus challenged with an injection of CT26 cells under the liver capsule before being treated 72 hours after inoculation and twice weekly for the duration of the experiment with intraperitoneal administrations of anti-CCL5 antibodies at the dose previously shown to be effective [20], [21]. Although 100% of the mice from both groups developed liver tumors, there was a significant reduction (30%) in the overall tumor load in the livers of the anti-CCL5-treated mice compared to the control group, as assessed by measuring liver weight (1.24 vs 1.78, P<0.05) (Figure 3B).

Besides liver tumors, we also observed differences in the extent of macrospcopic peritoneal carcinosis between the two groups of treatments (Figure 3C). Whereas peritoneal dissemination was found in 100% of the control mice, it was only observed in 40% of those treated with the anti-CCL5 antibodies.

### mPDGFRβ vaccination reduces growth of CT26 colon carcinoma metastases

**Figure 4:**
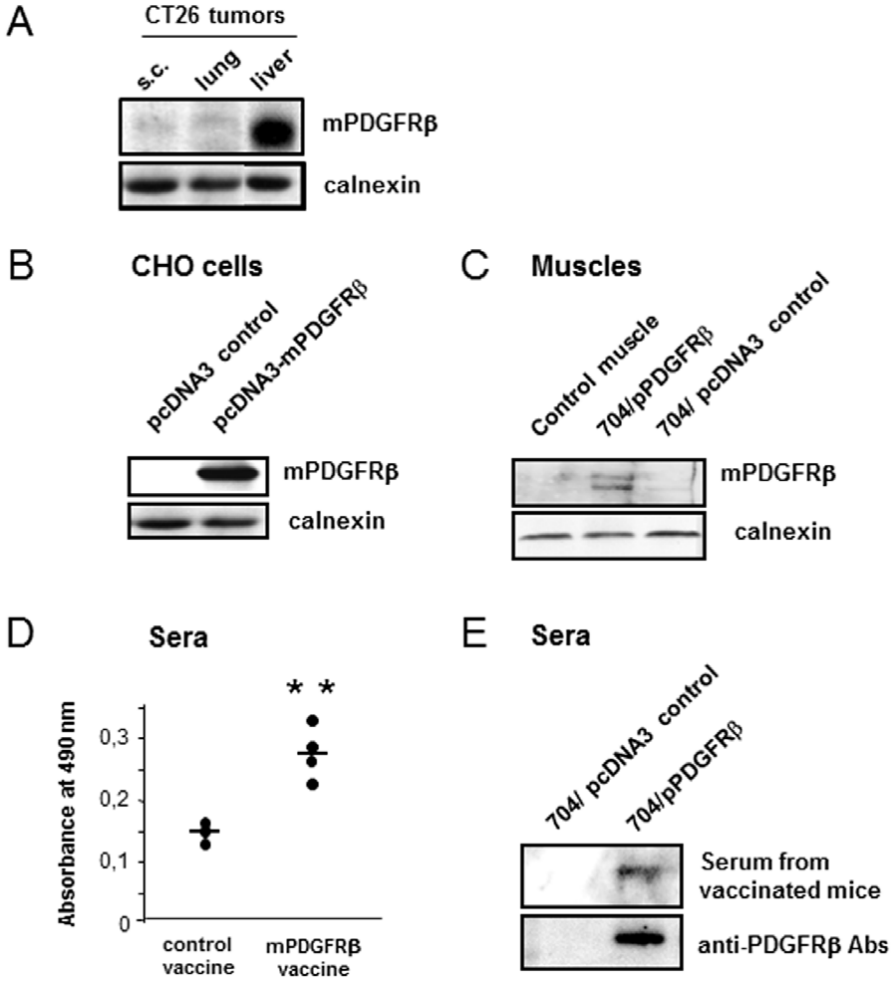
Formulation of DNA with 704 polymer increases mPDGFRβ expression level after intramuscular injection and induces a specific immune response. Western blot analyses of mPDGFRβ expression level (A) in CT26-derived subcutaneous tumors, pulmonary and liver metastases, (B) in pcDNA-3 control (with antisens PCR product) and pcDNA3-mPDGFRβ-transfected CHO cells and (C) in *tibialis anterior* muscles of Balb/c mice injected once with low-dose (25 µg) of mPDGFRβ encoding plasmid formulated in 704 copolymer (n = 4). (D) Determination of serum levels of mPDGFRβ-specific antibodies by ELISA. Mice were challenged on day 0, and restimulated on day +21, and day +42 with the DNA vaccine (50 µg) or the control vector formulated with 0.3% 704 before being sacrificed two weeks later for serum collection (n = 5). ** p<0.01. (E) Mouse sera were assayed for their ability to react with the band of the purified antigen from the mPDGFRβ-transfected CHO cells by Western blot analysis. Goat anti-mPDGFRβ antibodies from Santa Cruz were used for comparison.

**Figure 5:**
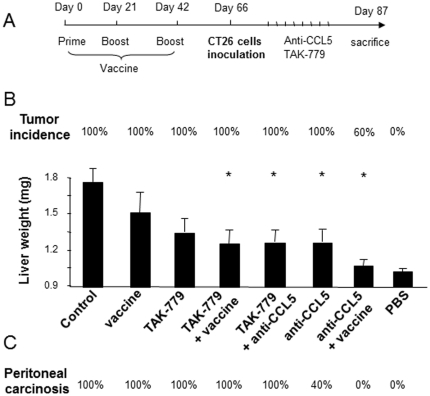
Combining CCL5- and PDGFRβ-directed strategies offers the greatest protection against liver tumors and peritoneal carcinosis. (A) Mice were challenged on day 0, and restimulated on day +21, and day +42 with the DNA vaccine (50 µg) or the control vector formulated with 0.3% poloxamine 704 before receiving three weeks later an injection of CT26 cells under the liver capsule. Seventy two hours later, mice were treated with CCL5-directed antibodies or with TAK-779 or both, as described in the Materials and Methods section. (B) Incidence and extent of tumor development within livers of CT26-challenged mice, immunized or not against PDGFRβ and treated either with anti-CCL5 antibodies, with TAK-779, or with both blockers. (C) Incidence of peritoneal carcinosis expressed as percent of mice bearing carcinosis. (n = 5/group). * p<0.05, ** p<0.01.

Given that the anti-CCL5 strategy could only lead to a moderate therapeutic effect against colon cancer, we next sought to evaluate the potential of CCL5 blockade administered in combination with an anti-PDGFRβ strategy. Indeed, the PDGF/PDGFRβ interaction is known to play a key role in the promotion of several malignancies including colorectal carcinoma. In particular, Wehler et al [30], have shown that PDGFRβ expression correlated with lymphatic dissemination in human colorectal cancer. Working on 99 human CRC biopsies, the authors reported by immunohistochemistry and RT-PCR that PDGFRβ expression occurred in 60% of the patients. Similarly, PDGFRβ expression was detected in four human CRC cell lines: Caco-2, HT29, SW480 and SW620. Consistent with this, we observed high levels of expression of mPDGFRβ in CT26 derived liver metastases (Figure 4A). As a consequence, we created a DNA vaccine encoding mPDGFRβ and verified the correct expression of mPDGFRβ by Western Blot analysis after transient transfection of CHO cells (Figure 4B). We then prepared formulations of DNA vaccine plasmids with 704, a tetrafunctionalized amphiphilic block copolymer that has previously been shown to improve intramuscular DNA vaccination by simultaneously increasing transgene expression and activating immunity [25], [26]. The potency of 704-formulated DNA vaccine plasmid to allow gene delivery into *tibialis anterior* muscles of 6 weeks-old Balb/c mice was evaluated by Western Blotting (Figure 4C) and confirmed muscular expression of the protein following administration of low-dose DNA (25 µg). According to a previously optimized immunization protocol [25], [26], mice were challenged on day 0, and restimulated on day +21, and day +42 with the DNA vaccine (50 µg) or the control vector formulated with 0.3% polymer 704. Two weeks after the prime-boost immunization, sera from both groups of animals were analysed for the presence of mPDGFRβ specific antibodies. As depicted on Figure 4D, immunized mice exhibited a humoral response to mPDGFRβ protein detected by ELISA (P = 0.005). The presence of specific mPDGFRβ antibodies in their sera was further confirmed by testing their ability to react with the band of the purified antigen from the mPDGFRβ-transfected CHO cells by Western blot analysis (Figure 4E) and by reprobing the blot with a commercial antibody specific to mPDGFRβ. The ability of our DNA-based vaccine to induce a mPDGFRβ-specific adaptive immune response led us to examine whether this approach could protect CT26-challenged mice. Following a prophylactic setting, we administered the mPDGFRβ vaccine according to the prime-boost regimen described above prior to inoculating the CT26 cells under the liver capsule (Figure 5A). Twenty-one days after tumor cell challenge, we compared the extent of tumor development within livers from both the immunized and the control animals. Although we observed a slight decrease (16%) in the tumor burdens of the mice vaccinated against mPDGFRβ compared to those of the non-immunized ones (1.49 vs 1.8) this reduction was not statistically significant (Figure 5B). In addition, the incidence of peritoneal carcinosis in vaccinated mice remained of 100% (Figure 5C).

### CCL5 blockade potentiates the targeting of mPDGFRβ in CT26 challenged mice

We next evaluated the impact of combined strategies on metastatic colon carcinoma. mPDGFRβ-vaccinated mice were thus challenged with CT26 cells before being treated with CCL5-directed antibodies. Mice treated with such combined regimen developed much smaller liver metastases compared to control animals (1.07 vs 1.79 in controls, P<0.05) (Figures 5B). In addition, vaccination allowed to increase the protective action of CCL5 blockade from 30% to 40% and to reduce tumor incidence from 100% to 60% in mice. Aside from reducing liver metastases, combined CCL5- and mPDGFRβ-directed strategies led to a drastic decrease in the macroscopic peritoneal dissemination in 100% of the mice whereas all other regimen failed to significantly impair carcinosis (Figure 5C).

In a next step, we attempted to disrupt CCL5 action using TAK-779, a non-peptide CCR5 antagonist. We therefore performed the systemic administration of TAK-779 into CT26-liver metastases bearing mice as previously described in pancreatic cancer [22]. However, TAK-779 treatment did not significantly protect mice from developing liver metastases (1.35 vs 1.79, P>0.05) and peritoneal dissemination was of 100% in those mice. Although, no significant difference was measured in the antitumor effect on the liver between TAK-779 and anti-CCL5 Abs (1.35 vs 1.23, P>0.05), there was a marked difference in the peritoneal carcinosis between both groups (100% with TAK-779 vs 40% with anti-CCL5). In addition, combining both treatments (anti-CCL5 Abs + TAK-779) failed to increase further the efficacy of CCL5 neutralization thus suggesting that blocking CCL5 action may recapitulate more signaling pathways than simply that of CCR5. Finally, the therapeutic efficacy of combining CCR5- and mPDGFRβ-directed strategies was much less than that of combining CCL5 Abs/vaccine in terms of tumor load (1.2 vs 1.07), of tumor incidence (100% vs 60%) and of peritoneal dissemination (100% vs 0%) again suggesting the superiority of CCL5 blockade + PDGFRβ vaccine over all other combinations (Figures 5 B and C).

### Remaining CT26 liver metastases in anti-CCL5 and anti-CCL5/PDGFRβ–treated animals display increased leukocytic infiltration

**Figure 6:**
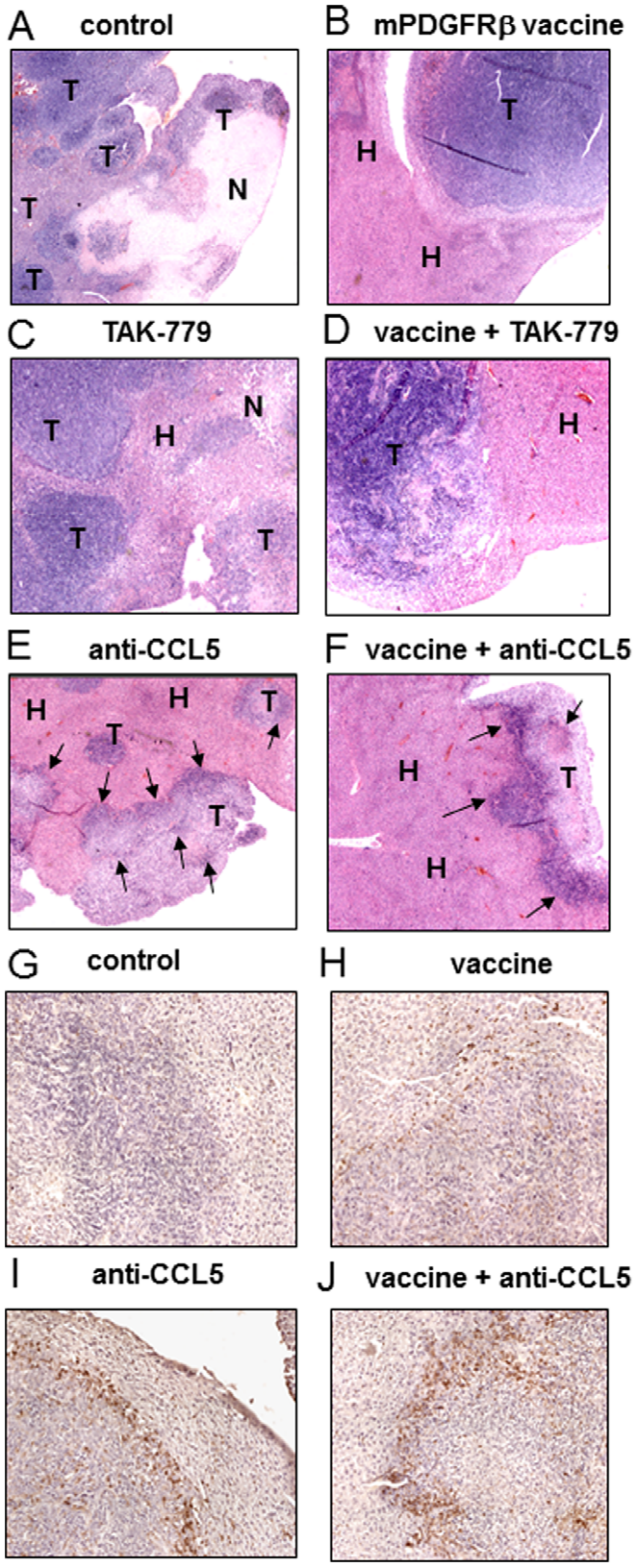
Liver histology at the time of sacrifice. (A–F) Liver histology was compared on sections of organs from CT26-challenged mice either untreated (A), or immunized with mPDGFRβ vaccine (B), or treated with TAK-779 (C), with TAK-779 plus mPDGFRβ vaccine (D), with anti-CCL5 antibodies (E) or with anti-CCL5 antibodies plus mPDGFRβ vaccine (F). T = liver tumors produced by CT26 cells; N = area of necrosis in a liver tumor; H = hepatocytes; Arrows indicate the presence of dense infiltrates at the interface with the healthy tissue and within lesions. (G–J) CD45-staining reveals immunoreactive leukocytes at the interface tumor-parenchyma and within hepatic lesions of anti-CCL5- (I) and anti-CCL5/PDGFRβ-treated mice (J) but less leukocytes within vaccinated mice (H) and almost no staining in untreated mice (G).

At a microscopic level, CT26 cells of control mice produced voluminous tumors composed of cells growing in interlacing bundles or sheets and displaying numerous mitotic figures. Necrotic areas were prominent in the largest tumors (Figure 6A). Livers from mice treated either with TAK-779, or with mPDGFRβ vaccine, or with both, displayed fewer nodules than those of untreated animals and most of these lesions were smaller in size (Figure 6 B, C and D). The most sizeable lesions resembled CT26 control tumors with areas of necrosis and minimal leukocytic infiltration. The surrounding parenchyma often displayed areas of remodelling. In contrast, mice receiving the anti-CCL5 antibodies either as single-agent treatment or in combination with the mPDGFRβ vaccine clearly developed much fewer and smaller lesions than all other groups (Figure 6 E and F). In addition, the rest of the parenchyma was comparable to that of normal animals. At the time of sacrifice, lesions often featured necrotic tumor cells admixed with dense infiltrates. The most remarkable observations concerned small and medium sized nodules showing at their periphery the presence of bands of cells with dense round nuclei. CD45 immunostaining confirmed the presence of immunoreactive leukocytes at the interface with the healthy tissue and within the lesions (Figure 6 I and J) of liver sections from the anti-CCL5- and combined regimen-treated mice whereas considerably less CD45-positive leukocytes were detected on liver sections from control and vaccinated animals (Figure 6 G and H).

## Discussion

In the present work, we provide the first evidence of the implication of CCL5 in the pathophysiology of colorectal carcinoma. CCL5 neutralization resulted in reducing the extent of development of experimental colon tumors implanted into mice either subcutaneously or under the liver capsule, and also led to decrease the peritoneal carcinosis in those mice. With regard to hepatic metastases, this result is consistent with the fact that metastatic liver resection pieces exhibited the highest levels of expression for CCL5, as well as for its receptors CCR1 and CCR5. Additional evidence supporting the involvement of the CCL5/CCR5 axis in colon cancer progression is provided by the antitumor effect of TAK-779, the CCR5 antagonist. Proliferative and migratory responses of the tumor cells from human and mouse origins to CCL5 were significantly reduced by TAK-779, thus implicating CCR5-mediated processes in the direct stimulatory effects of CCL5 onto CRC cells. In addition, the fact that only partial inhibitory activity of TAK-779 against CRC cells *in vitro* was observed suggested the involvement of CCR5-independent mechanisms. Consistent with the redundancies that exist in the chemokine family, CCL5 acts through two additional G-protein coupled receptors termed CCR1 and CCR3 [31]. It is therefore likely that some of the tumor-promoting properties of CCL5 in colon cancer could be mediated through one or two of those additional receptors. Accordingly, the protective effect of TAK-779 against tumor development in mice was much less pronounced than that of CCL5 blockade. In this regard, our data contrast with those obtained in breast and pancreatic cancers where CCR5- and CCL5-directed treatments were equally efficient in reducing tumor progression. Collectively, our observations argue for a significant implication of CCL5 in the growth and spread of CRC cells in vivo and in vitro, whereas CCR5 signaling appears to be involved to a much lesser extent in the pathogenesis of colorectal cancer.

Aside from being a promalignant factor acting directly onto colon cancer cells, CCL5 also appeared in our studies as a regulator of inflammatory infiltrates within tumor tissues. Indeed, our data indicate that administration of CCL5-directed antibodies into mice led to an accumulation of leukocytes within the cancer stroma as well as at the interface tumor-parenchyma. In contrast to the abundancy of inflammatory infiltrates observed in the lesions of the anti-CCL5-treated animals, such responses were rather poor wihin liver tumors of other treated mice, suggesting that CCL5 blockade may favour immune destruction of the tumor. According to a recent study by Tan et al., CCL5 is able to induce the recruitment of regulatory T cells into pancreatic tumors and therefore to actively induce immune tolerance [22]. With regard to immune escape mechanisms, cancer cells exposed to CCL5 have also been shown to induce apoptosis of CD8+ T cells in gastric cancer [20]. The involvement of additional cell populations in the promalignant action of CCL5 is also suggested by our quantitative real time-PCR data performed with HT29 xenografts implanted into SCID mice. Indeed, the fact that CCR5 overexpression was observed within human CRC biopsies and CT26 tumors using primers recognizing both tumor cells and host cells but was not detected within human HT29 cells *in vivo* indicates that CCR5 expression may originate from host cells in the SCID model. Although SCID mice lack a functioning immune system, they do have macrophages. In addition, stromal cells such as fibroblasts or vascular smooth muscle cells, as well as endothelial cells also express CCR1, CCR3 and CCR5 and may thus participate in CCL5 promalignant action. The tumor promoting activity of fibroblasts in colon cancer has clearly been established [32]. As per macrophages, increasing infiltrates have been observed with advanced stages of colorectal carcinoma [33]. However, according to the “macrophage balance hypothesis” described by Mantovani [34], the exact role played by macrophages can vary from tumor suppression to promotion depending on their number and state of activation. Therefore, even if the aforementioned mechanisms are probably not exclusive, it is possible that simultaneously eliciting immune responses and compromising tumor-promoting activities through CCL5 neutralization may account for the reduced outgrowth of colon tumor cells in vivo.

Although, further studies will be necessary to dissect the precise contribution of diverse factors such as the local environment or the immune system in the tumor promoting action of CCL5, our data clearly indicate that interfering with CCL5 signaling appears as an attractive approach to offer protection against CRC.

Beside its potential as single agent treatment, CCL5 blockade has recently been reported to potentiate chemotherapy. In the present study, we have investigated whether anti-CCL5 antibodies could potentiate a mPDGFRβ-directed approach, targeting this receptor being a strategy currently tested in the clinic (through compounds such as Imatinib, Nilotinib or Sunitinib) for the treatment of various oncological indications including colorectal carcinoma (melanoma, sarcoma, breast, lung and prostate cancer [35]). Aside from inhibitors, preclinical approaches have also been conducted to develop PDGFRβ-based DNA vaccine aiming at preventing tumor development [36]–[40]. In this context, Kaplan and colleagues have demonstrated that immunizing mice with an orally delivered mPDGFRβ-based DNA vaccine (i.e. gavage with attenuated *Salmonella typhimurium* bacteria) offered significant protection against the growth and dissemination of murine colon cancer. Here, we have assessed the potential of a mPDGFRβ encoding DNA vaccine formulated with the amphiphilic block copolymer 704. As recently reported, this DNA vaccine strategy is indeed very attractive due to its ability to elicit a significant immune response with 50 times less encoding DNA than other genetic vaccination vectors but also due to its high safety index [25], [26]. In addition, immunizing mice against a model antigen using such DNA/polymer vaccine approach was shown to protect them against tumor challenge in an orthotopic hepatocellular carcinoma model [25], [26]. Up to now, this strategy has however not been exploited to trigger an immune response against a self-antigen such as PDGFRβ. In the present study, we observed that the prophylactic immunization performed to trigger PDGFRβ–specific immunotherapy in the CT26 colon cancer model only led to a minor reduction in liver metastasis compared to control animals thus suggesting that improvements in the vaccine protocol have to be envisaged for its application as single agent strategy. Interestingly however, immunization against PDGFRβ offered a drastic protection when combined to anti-CCL5 antibodies. Although the effect was not total on liver metastases, only 60% of the mice developed tumors after the combination regimen (vaccine plus CCL5-directed approaches), thus supporting a cumulative effect of both strategies. Of particular interest, 100% of the mice were also protected from peritoneal carcinosis. In contrast, combining TAK-779 treatment with PDGFRβ vaccine failed to considerably reduce the extent of tumor and carcinosis development compared to each treatment alone, again supporting the greatest potential of CCL5 inhibition compared to CCR5 antagonism in this CRC model. Previous studies from the literature have described an up-regulation of mPDGFRβ expression within the tumor stroma of several neoplasms including colon carcinoma [41]–[44] in particular on the tumor-associated pericytes and fibroblasts [45]. Moreover, PDGFRβ signaling has been shown to stimulate recruitment of pericytes and their coverage of blood vessels [46]–[48]. Accordingly, PDGFRβ-directed treatments, such as STI571 or DNA vaccines, have led to apoptosis of tumor-associated pericytes and endothelial cells and thus to confine the vasculature to an immature stage in colon tumors [36], [37]. It is therefore possible that simultaneous disruption of CCL5/CCRs axes potentiates this effect on the vasculature by reducing the immune tolerance towards tumor cells. Consistent with this hypothesis, we found increased leukocytic infiltrates within lesions of immunized mice after treatment with anti-CCL5 antibodies. In summary, our data point to a multiple contribution of CCL5 in colon cancer development, including its ability to promote metastatic features of tumor cells and to dampen anti-tumor immunity. In addition, the multiplicity of promalignant signals and of tumor-promoting cell types within colon tumors mandates therapy combining multiple inhibitions directed against both cancer and stromal cells to produce an effective therapy of the neoplasm.

Taken together, our findings show that interfering with CCL5 signaling may be an approach to control the progression of this malignancy, alone or in combination with mPDGFRβ-directed therapies.

## Supporting Information

Table S1 **ΔCT: CT(target) – CT(actin); Fold: fold change in mRNA expression between tumor and healthy tissues; P: p values considered significant when P<0.05.**
(PPT)

## References

[1] Mueller MM, Fusenig NE (2004). Friends or foes - bipolar effects of the tumour stroma in cancer.. Nat Rev Cancer.

[2] Bhowmick NA, Neilson EG, Moses HL (2004). Stromal fibroblasts in cancer initiation and progression.. Nature.

[3] Tlsty TD, Coussens LM (2006). Tumor stroma and regulation of cancer development.. Annu Rev Pathol.

[4] Ito M, Yoshida K, Kyo E, Ayhan A, Nakayama H (1990). Expression of several growth factors and their receptor genes in human colon carcinomas.. Virchows Arch B Cell Pathol Incl Mol Pathol.

[5] De Jong KP, Stellema R, Karrenbeld A, Koudstaal J, Gouw AS (1998). Clinical relevance of transforming growth factor alpha, epidermal growth factor receptor, p53, and Ki67 in colorectal liver metastases and corresponding primary tumors.. Hepatology.

[6] Hemming AW, Davis NL, Kluftinger A, Robinson B, Quenville NF (1992). Prognostic markers of colorectal cancer: an evaluation of DNA content, epidermal growth factor receptor, and Ki-67.. J Surg Oncol.

[7] Kuwai T, Kitadai Y, Tanaka S, Onogawa S, Matsutani N (2003). Expression of hypoxia-inducible factor-1alpha is associated with tumor vascularization in human colorectal carcinoma.. Int J Cancer.

[8] Gschwind A, Fischer OM, Ullrich A (2004). The discovery of receptor tyrosine kinases: targets for cancer therapy.. Nat Rev Cancer.

[9] Balkwill F (2004). Cancer and the chemokine network.. Nat Rev Cancer.

[10] Erreni M, Bianchi P, Laghi L, Mirolo M, Fabbri M (2009). Expression of chemokines and chemokine receptors in human colon cancer.. Methods Enzymol.

[11] Cambien B, Karimdjee BF, Richard-Fiardo P, Bziouech H, Barthel R (2009). Organ-specific inhibition of metastatic colon carcinoma by CXCR3 antagonism.. Br J Cancer.

[12] Pradelli E, Karimdjee-Soilihi B, Michiels JF, Ricci JE, Millet MA (2009). Antagonism of chemokine receptor CXCR3 inhibits osteosarcoma metastasis to lungs.. Int J Cancer.

[13] Luboshits G, Shina S, Kaplan O, Engelberg S, Nass D (1999). Elevated expression of the CC chemokine regulated on activation, normal T cell expressed and secreted (RANTES) in advanced breast carcinoma.. Cancer Res.

[14] Duell EJ, Casella DP, Burk RD, Kelsey KT, Holly EA (2006). Inflammation, genetic polymorphisms in proinflammatory genes TNF-α, RANTES, and CCR5, and risk of pancreatic adenocarcinoma.. Cancer Epidemiol Biomarkers Prev.

[15] Tsukishiro S, Suzumori N, Nishikawa H, Arakawa A, Suzumori K (2006). Elevated serum RANTES levels in patients with ovarian cancer correlate with the extent of the disorder.. Gynecol Oncol.

[16] Niwa Y, Akamatsu H, Niwa H, Sumi H, Ozaki Y (2001). Correlation of tissue and plasma RANTES levels with disease course in patients with breast or cervical cancer.. Clin Cancer Res.

[17] Kim HK, Song KS, Park YS, Kang YH, Lee YJ (2003). Elevated levels of circulating platelet microparticles, VEGF, IL-6 and RANTES in patients with gastric cancer: possible role of a metastasis predictor.. Eur J Cancer.

[18] Vaday GG, Peehl DM, Kadam PA, Lawrence DM (2006). Expression of CCL5 (RANTES) and CCR5 in prostate cancer.. Prostate.

[19] Yaal-Hahoshen N, Shina S, Leider-Trejo L, Barnea I, Shabtai EL (2006). The chemokine CCL5 as a potential prognostic factor predicting disease progression in stage II breast cancer patients.. Clin Cancer Res.

[20] Sugasawa H, Ichikura T, Kinoshita M, Ono S, Majima T (2008). Gastric cancer cells exploit CD4+ cell-derived CCL5 for their growth and prevention of CD8+ cell-involved tumor elimination.. Int J Cancer.

[21] Karnoub AE, Dash AB, Vo AP, Sullivan A, Brooks MW (2007). Mesenchymal stem cells within tumour stroma promote breast cancer metastasis.. Nature.

[22] Tan MC, Goedegebuure PS, Belt BA, Flaherty B, Sankpal N (2009). Disruption of CCR5-dependent homing of regulatory T cells inhibits tumor growth in a murine model of pancreatic cancer.. J Immunol.

[23] Zhang X, Haney KM, Richardson AC, Wilson E, Gewirtz DA (2010). Anibamine, a natural product CCR5 antagonist, as a novel lead for the development of anti-prostate cancer agents.. Bioorg Med Chem Lett.

[24] Horst D, Budczies J, Brabletz T, Kirchner T, Hlubek F (2009). Invasion associated up-regulation of nuclear factor kappaB target genes in colorectal cancer.. Cancer.

[25] McIlroy D, Barteau B, Cany J, Richard P, Gourden C (2009). DNA/amphiphilic block copolymer nanospheres promote low-dose DNA vaccination.. Mol Ther.

[26] Cany J, Barteau B, Tran L, Gauttier V, Archambeaud I (2010). AFP-specific immunotherapy impairs growth of autochthonous hepatocellular carcinoma in mice.. J Hepatol.

[27] Vitale S, Cambien B, Karimdjee BF, Barthel R, Staccini P (2007). Tissue-specific differential antitumour effect of molecular forms of fractalkine in a mouse model of metastatic colon cancer.. Gut.

[28] Baba M, Nishimura O, Kanzaki N, Okamoto M, Sawada H (1999). A small-molecule, nonpeptide CCR5 antagonist with highly potent and selective anti-HIV-1 activity.. Proc Natl Acad Sci U S A.

[29] Marella M, Chabry J (2004). Neurons and astrocytes respond to prion infection by inducing microglia recruitment.. J Neurosci.

[30] Wehler TC, Frerichs K, Graf C, Drescher D, Schimanski K (2008). PDGFRalpha/beta expression correlates with the metastatic behavior of human colorectal cancer: a possible rationale for a molecular targeting strategy.. Oncol Rep.

[31] Tanaka T, Bai Z, Srinoulprasert Y, Yang BG, Hayasaka H (2005). Chemokines in tumor progression and metastasis.. Cancer Sci.

[32] Elenbaas B, Weinberg RA (2001). Heterotypic signaling between epithelial tumor cells and fibroblasts in carcinoma formation.. Exp Cell Res.

[33] Bailey C, Negus R, Morris A, Ziprin P, Goldin R (2007). Chemokine expression is associated with the accumulation of tumour associated macrophages (TAMs) and progression in human colorectal cancer.. Clin Exp Metastasis.

[34] Mantovani A, Bottazzi B, Colotta F, Sozzani S, Ruco L (1992). The origin and function of tumor-associated macrophages.. Immunol Today.

[35] Grimminger F, Schermuly RT, Ghofrani HA Targeting non-malignant disorders with tyrosine kinase inhibitors.. Nat Rev Drug Discov.

[36] Buchdunger E, Cioffi CL, Law N, Stover D, Ohno-Jones S (2000). Abl protein-tyrosine kinase inhibitor STI571 inhibits in vitro signal transduction mediated by c-kit and platelet-derived growth factor receptors.. J Pharmacol Exp Ther.

[37] Kim R, Emi M, Arihiro K, Tanabe K, Uchida Y (2005). Chemosensitization by STI571 targeting the platelet-derived growth factor/platelet-derived growth factor receptor-signaling pathway in the tumor progression and angiogenesis of gastric carcinoma.. Cancer.

[38] Kitadai Y, Sasaki T, Kuwai T, Nakamura T (2006). Targeting the expression of platelet-derived growth factor receptor by reactive stroma inhibits growth and metastasis of human colon carcinoma.. Am J Pathol.

[39] Kuwai T, Nakamura T, Sasaki T, Kitadai Y (2008). Targeting the EGFR, VEGFR, and PDGFR on colon cancer cells and stromal cells is required for therapy.. Clin Exp Metastasis.

[40] Kaplan CD, Kruger JA, Zhou H, Luo Y, Xiang R (2006). A novel DNA vaccine encoding PDGFRbeta suppresses growth and dissemination of murine colon, lung and breast carcinoma.. Vaccine.

[41] Coltrera MD, Wang J, Porter PL, Gown AM (1995). Expression of platelet-derived growth factor B-chain and the platelet-derived growth factor receptor beta subunit in human breast tissue and breast carcinoma.. Cancer Res.

[42] Bhardwaj B, Klassen J, Cossette N, Sterns E, Tuck A (1996). Localization of platelet-derived growth factor beta receptor expression in the periepithelial stroma of human breast carcinoma.. Clin Cancer Res.

[43] Kawai T, Hiroi S, Torikata C (1997). Expression in lung carcinomas of platelet-derived growth factor and its receptors.. Lab Invest.

[44] Singer CF, Hudelist G, Lamm W, Mueller R, Czerwenka K (2004). Expression of tyrosine kinases in human malignancies as potential targets for kinase-specific inhibitors.. Endocr Relat Cancer.

[45] Ostman A, Heldin CH (2001). Involvement of platelet-derived growth factor in disease: development of specific antagonists.. Adv Cancer Res.

[46] Risau W, Drexler H, Mironov V, Smits A, Siegbahn A (1992). Platelet-derived growth factor is angiogenic in vivo.. Growth Factors.

[47] Ostman A (2004). PDGF receptors-mediators of autocrine tumor growth and regulators of tumor vasculature and stroma.. Cytokine Growth Factor Rev.

[48] Bergers G, Song S, Meyer-Morse N, Bergsland E, Hanahan D (2004). Benefits of targeting both pericytes and endothelial cells in the tumor vasculature with kinase inhibitors.. J Clin Invest.

