# Supplementary figures and images for: CCL5 Neutralization Restricts Cancer Growth and Potentiates the Targeting of PDGFRβ in Colorectal Carcinoma
Source: PLoS One. 2011 Dec 20;6(12):e28842. doi: 10.1371/journal.pone.0028842 (PMC3243667; doi:10.1371/journal.pone.0028842)

## Slide 1
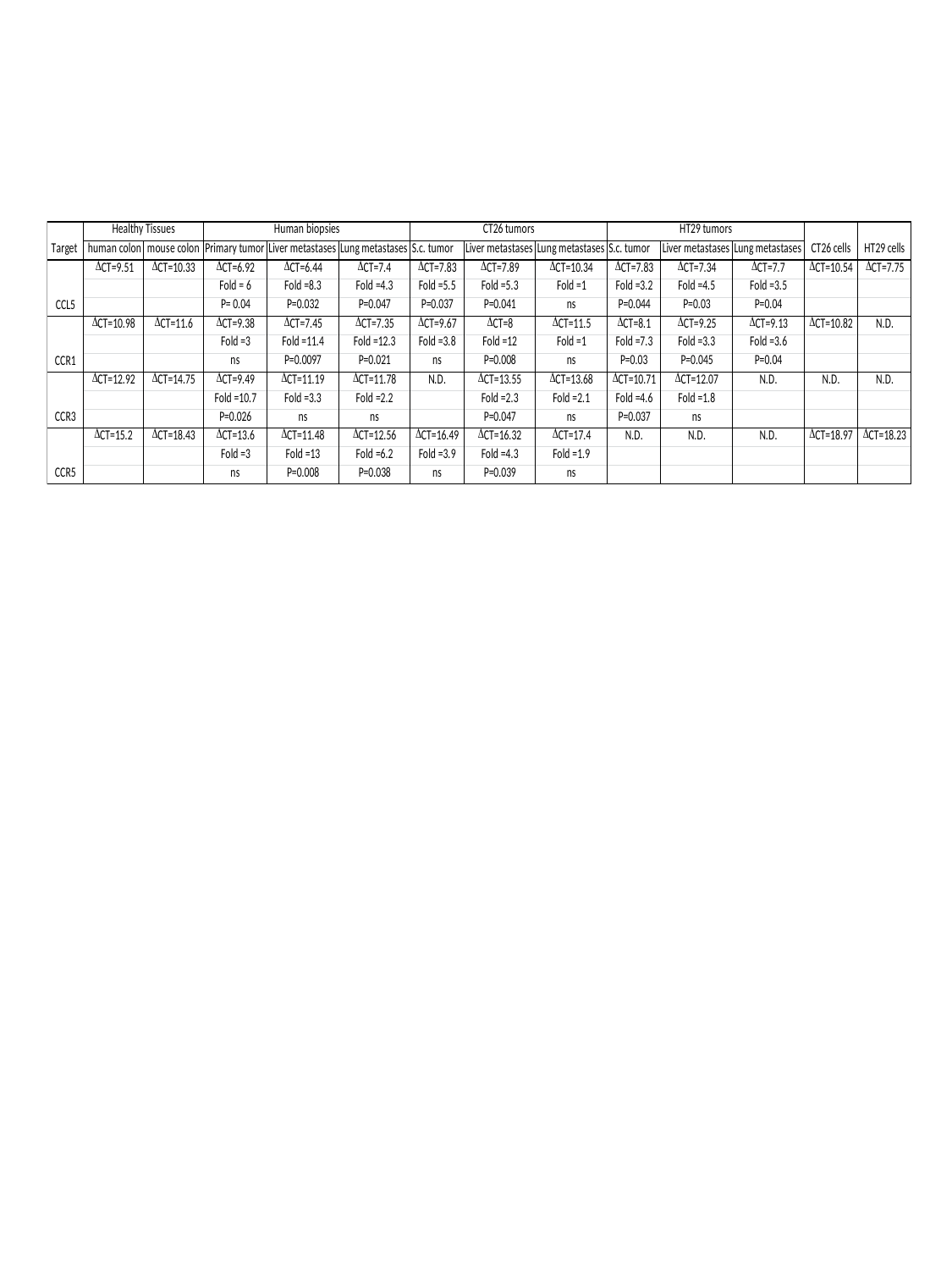

Supplement: Table S1 — ΔCT: CT(target) – CT(actin); Fold: fold change in mRNA expression between tumor and healthy tissues; P: p values considered significant when P<0.05. (PPT) [file pone.0028842.s001.ppt]
